# Evaluation of operating room reverse Trendelenburg positioning and its effect on postoperative hypoxemia, aspiration, and length of stay: a retrospective study of consecutive patients

**DOI:** 10.1186/s13741-017-0067-2

**Published:** 2017-08-22

**Authors:** C. Michael Dunham, Barbara M. Hileman, Amy E. Hutchinson, Tamara Antonaccio, Elisha A. Chance, Gregory S. Huang, Gregory Szmaj, Kristen Calabro, Cynthia Bishop, Tyson T. Schrickel

**Affiliations:** 1 Trauma, Critical Care, and General Surgery Services, St. Elizabeth Youngstown Hospital, 1044 Belmont Ave., Youngstown, OH 44501 USA; 2 Trauma and Neurosciences Research Department, St. Elizabeth Youngstown Hospital, 1044 Belmont Ave., Youngstown, OH 44501 USA; 3 Department of Anesthesiology, St. Elizabeth Youngstown Hospital, 1044 Belmont Ave., Youngstown, OH 44501 USA; 4 OR Nursing Staff, St. Elizabeth Youngstown Hospital, 1044 Belmont Ave., Youngstown, OH 44501 USA; 5 Department of Surgery, St. Elizabeth Youngstown Hospital, 1044 Belmont Ave., Youngstown, OH 44501 USA; 6 Surgical and Perioperative Services, St. Elizabeth Youngstown Hospital, 1044 Belmont Ave., Youngstown, OH 44501 USA; 7 Department of Orthopedic Surgery, St. Elizabeth Youngstown Hospital, 1044 Belmont Ave., Youngstown, OH 44501 USA

**Keywords:** Aspiration, respiratory, Hypoxemia, Period, perioperative, Operating rooms, Supine position, Reverse Trendelenburg

## Abstract

**Background:**

In 2014, this group published an investigation of surgical patients from 2012 who had substantial rates of postoperative hypoxemia (POH) and perioperative pulmonary aspiration (POPA). Therefore, we investigated whether intraoperative reverse Trendelenburg positioning (RTP) decreases POH and POPA rates.

**Methods:**

Consecutive ASA I–IV surgical patients who had preoperative pulmonary stability requiring general anesthesia with endotracheal intubation were evaluated. Using pulse oximetry, hypoxemia was documented intraoperatively and during the 48 h following PACU discharge. POPA was the presence of a pulmonary infiltrate with hypoxemia. In early 2015, a multifaceted effort was undertaken to enhance anesthesiologist and operating nurse awareness of RTP to potentially decrease POH and POPA rates. Analyses included (1) combining 2012 and 2015 cohorts to assess risk conditions, (2) comparing post-campaign 2015 (increased RTP) and 2012 cohorts, and (3) comparing 2015 patients with audit-documented RTP during surgery to the other 2015 patients.

**Results:**

Combining the 500 patients in 2012 with the 1000 in 2015 showed that POH had increased mortality (2.3%), compared to no POH (0.2%; *p* = 0.0004). POH had increased postoperative length of stay (LOS) (4.6 days), compared to no POH (2.0 days; *p* < 0.0001). POPA had increased mortality (7.7%) and LOS (8.8 days), compared to no POPA (0.4%; *p* = 0.0004; 2.3 days; *p* < 0.0001). Open aortic, cranial, laparotomy, and neck procedures had greater POH (41.3%) and LOS (4.0 days), compared to other procedures (16.3%; *p* < 0.0001; 2.2 days; *p* < 0.0001). Glycopyrrolate on induction had lower POH (17.4%) and LOS (1.9 days), compared to no glycopyrrolate (21.6%; *p* = 0.0849; 2.7 days; *p* < 0.0001). POH was lower (18.1%) in 2015, than in 2012 (25.6%; *p* = 0.0007). POPA was lower with RTP in 2015 (0.6%), than in 2012 (4.8%; *p* = 0.0088). For the 2015 patients, LOS was lower with audit-documented RTP (2.2 days), compared to other patients (2.7 days; *p* = 0.0246).

**Conclusions:**

These findings are only hypothesis-generating. A randomized clinical trial is needed to confirm whether RTP has an inverse association with POH and POPA, and if RTP and glycopyrrolate are associated with improved outcomes.

**Trial registration:**

ClinicalTrials.gov, NCT02984657

## Background

In 2014, our group published an investigation of patients who underwent extra-thoracic procedures under general anesthesia and had preoperative pulmonary stability (Dunham et al. 2014). We provided evidence to suggest that the substantial rates of perioperative hypoxemia and perioperative pulmonary aspiration (POPA) may have been related to horizontal recumbency.

It has been established that POPA can lead to death (Cotton and Smith 1984; Morgan 1984; Tiret et al. 1986; Kozlow et al. 2003) and may cause clinically significant morbidities (Cotton and Smith 1984; Kozlow et al. 2003; Kluger and Short 1999). Of importance, there are findings from operating room (OR), intensive care unit, and animal investigations demonstrating that aspiration can occur despite the presence of a cuffed endotracheal tube (Ewig and Torres 2002; Torres et al. 1992; Reali-Forster et al. 1996; Petring et al. 1986; Seegobin and van Hasselt 1986; Kalinowski and Kirsch 2004). Further, clinical evidence demonstrates that horizontal positioning in mechanically ventilated patients is a risk for pulmonary aspiration with lung inflammation (Kalinowski and Kirsch 2004; Smith and Ng 2003) and ventilator-associated pneumonia (Ewig and Torres 2002; Torres et al. 1992; Ferrer and Artigas 2002; Keenan et al. 2002; Koeman et al. 2001; Fernandez-Crehuet et al. 1997; Drakulovic et al. 1999; Kollef 1993). Accordingly, the Institute for Healthcare Improvement recommends elevating the head of the bed during intensive care unit mechanical ventilation to prevent pulmonary aspiration and ventilator-associated pneumonia (Institute for Healthcare Improvement 2012).

Of relevance, previous literature indicates that patients undergoing general endotracheal anesthesia for a surgical procedure are primarily placed in a supine, lithotomy, lateral, or prone position (Blitt et al. 1970; McEwen 1996; Adedeji et al. 2010), where horizontal recumbency is typically enforced (McEwen 1996; Adedeji et al. 2010; Smith 1990). Horizontal recumbency for typical operative body positions is promulgated within the operative nursing literature and teaching circles as common practice (McEwen 1996; Adedeji et al. 2010; Smith 1990). Specifically, horizontal positioning is disseminated by the use of specific narrative description statements (McEwen 1996; Adedeji et al. 2010) and inclusion of illustrations and photographs (McEwen 1996; Smith 1990; Mulier et al. 2010) that portray horizontal recumbency. Four review publications about POPA were evaluated for comments regarding body positioning. The most current review only included a single comment regarding body positioning (Abdulla 2013) and another did not mention body positioning (Engelhardt and Webster 1999). Ng et al. indicated that the Trendelenburg position is a risk for POPA, and lithotomy positioning may be a risk for POPA (Ng and Smith 2001). The greatest attention to body position as a risk for POPA was in a review by Kalinowski et al. in 2004 (Kalinowski and Kirsch 2004). The authors stated that aspiration is common in patients with impaired consciousness in the supine position, and with successful tracheal intubation, pulmonary aspiration seems less frequent if the patient’s head is elevated 45° (Kalinowski and Kirsch 2004). When considering reported evidence for the risks for POPA, horizontal recumbency, as a common practice, is counterintuitive.

In a previous work, we reported that the perioperative hypoxemia rate was substantial in all operative procedure categories (Dunham et al. 2014). Although the primary body position during the operative procedure was supine or lithotomy, the standard anesthesia practice was to maintain horizontal recumbency in all patients, except for a few in the sitting position. The previous study findings and literature documentation were consistent with the notion that perioperative hypoxemia, in part, may be a manifestation of occult-pulmonary or micro-pulmonary aspiration during horizontal recumbency.

In response to the earlier study findings and considerations from relevant literature, the primary purpose of the current study was to investigate whether reverse Trendelenburg positioning (RTP) during general anesthesia may have an inverse association with postoperative hypoxemia (POH) and POPA. Specifically, the study institution implemented an endeavor in 2015 to increase RTP awareness as a practice that may be beneficial. The authors compared 500 consecutive surgical patients from the previous investigation in 2012 (with decreased RTP awareness) to a cohort of 1000 consecutive surgical patients in 2015 (with improved RTP awareness). It was hypothesized that RTP would be associated with lower POH and POPA rates.

## Methods

The analyses included two cohorts: patients who met the inclusion criteria in 2012 and patients who met the same inclusion criteria in 2015 following OR nursing and anesthesiology staff RTP awareness training. The primary goal was to compare patients in 2012 (with decreased RTP awareness) to patients in 2015 (with improved RTP awareness). The secondary goal was to combine the patient cohorts in 2012 and 2015 and describe clinical associations with mortality, POH, POPA, and length of stay (LOS).

The present study was approved by the Mercy Health Youngstown Institutional Review Board for human investigations (15–023 approved July 16, 2015). The need for written informed consent was waived, because of the study’s retrospective nature. Patients’ records and information were anonymized and de-identified before analysis.

### Inclusion criteria

Inclusion criteria were patients aged 18 years or older, those who had preoperative pulmonary stability, and patients who underwent an operative procedure that required endotracheal intubation and a general anesthetic. Preoperative pulmonary stability criteria included a respiratory rate of 12–24 breaths per minute, and either an oxygen saturation measure by pulse oximetry (SpO_2_) ≥ 94% with room air or nasal cannula oxygen with a flow rate of 1 to 2 L/min or partial pressure of oxygen/inspired oxygen (PaO_2_/FiO_2_) ≥ 300, if greater supplemental oxygen was received. Patients were identified through the surgery case log, and data were collected from the electronic medical record (EMR).

### Exclusion criteria

Exclusion criteria were tracheal intubation before emergency department arrival, endotracheal intubation > 24 h before surgery, thoracotomy procedure, any cardiac procedure, Glasgow Coma Scale score < 13, an American Society of Anesthesiologists (ASA) classification V or VI, and patients with more than one surgery requiring tracheal intubation during the same hospitalization.

### Preoperative and intraoperative variables

Preoperative variables were age, ASA classification, emergency versus elective procedures, preexisting lung disease (PELD), and weight. PELD was considered present when a patient required any of the following respiratory therapies on a daily basis: home bi-level positive airway pressure, supplemental oxygen, an inhalational bronchodilator, or systemic bronchodilator or steroid. This information was ascertained by reviewing the anesthesia preoperative assessment note and the history and physical examination documented in each patient’s EMR. Intraoperative variables were OR duration (OR minutes), operative procedure categories, glycopyrrolate administration during anesthesia induction, and amount of fluid input. These data were gathered from the anesthesia intraoperative record, which had been hand written and scanned into the electronic medical record.

### Hypoxemia classification

As perioperative pulse oximetry monitoring is routine at our institution, we used intraoperative hypoxemia (IOH) and/or POH as a potential indicator of POPA. A co-investigator examined each patient’s anesthesia operative record and documented the presence of intraoperative SpO_2_ < 98%. A co-investigator also screened the EMR for evidence of POH. A positive POH screen was defined as two or more episodes of SpO_2_ < 94%, on room air or nasal cannula supplemental oxygen at 1–2 L/min, or SpO_2_ < 98% with greater supplemental oxygen within a 24-h period, during the 48 h following surgery. SpO_2_ < 94% during the first 2 h following OR extubation was not counted as a POH event, as hypoventilation may be related to post-anesthesia recovery. The first author, a retired board-certified surgical intensivist, reviewed each patient’s data whenever a patient had IOH and/or a positive POH screen. FiO_2_ was typically 60–100%. Whenever the intraoperative SpO_2_ was clearly < 98% and the intraoperative FiO_2_ was subsequently increased, the patient was classified as having an episode of IOH. When the postoperative SpO_2_ had decreased by ≥ 5% compared to the preoperative value, the patient was categorized as having an episode of POH.

There was no automated SpO_2_ data inflow into the electronic medical record (neither in the OR nor outside the OR). Within the OR, any decrement in SpO_2_ prompted an investigation that the pulse oximetry probe had proper patient contact. Outside the OR, SpO_2_ probe contact was confirmed by the nurse or respiratory therapist and directly entered into the electronic medical record by the nurse or respiratory therapist.

### POPA classification

POPA was defined as the presence of an IOH or POH event and an acute pulmonary infiltrate on thoracic radiographic imaging (chest radiograph or computed tomography scan) within the 48-h period following surgery. The first author examined each chest radiographic image available in the EMR in patients categorized with IOH or POH, for a pulmonary infiltrate. When the first author’s findings of an infiltrate were corroborated by the radiologist’s report, the patient was classified as POPA-positive.

### Selection of the RTP target

In early 2015, a literature search for RTP intervention studies identified 18 publications. Of these studies, seven (39%) used an RTP target of 10°, whereas the other studies had five other RTP target levels (5–30°), with a frequency of ≤ 17% each. Following this literature review, the first author and directors of OR nursing and anesthesiology assembled in the OR. The first author was positioned on the OR table, and RTP was adjusted to a level where the chief of anesthesiology believed that the risk for pulmonary aspiration would likely be mitigated. The OR table angle was 8.3°, as measured using a digital protractor (model 320.48295; Craftsman® Digital Torpedo Level, Hoffman Estates, IL). On the basis of this experience and the literature review, we selected a 10° tilt as the RTP target.

### Enhancing staff RTP awareness

In early 2015, the director of surgical and perioperative services, the manager and assistant manager of the OR nursing staff, and the chief of anesthesiology endorsed the view that RTP may mitigate POH and POPA. Their support was based on an appraisal of our publication findings from 2014, a review of the relevant literature, and the recommendation by the Institute for Healthcare Improvement.

In early April 2015, formal educational presentations regarding OR RTP were provided to the OR nursing staff, attending anesthesiologists and certified registered nurse anesthetists, general surgical attendings and residents, and orthopedic surgical attendings and residents. The presentations included (1) a literature review demonstrating that aspiration occurs despite the use of cuffed endotracheal tubes, (2) a literature review showing that horizontal positioning is associated with pulmonary aspiration in mechanically ventilated patients, (3) the Institute for Healthcare Improvement recommendation, (4) a review of our study published in 2014 regarding perioperative hypoxemia and aspiration, and (5) pictorials illustrating 10° RTP using a commercial OR table protractor. Additionally, discussions regarding RTP pros and cons took place between the first author and chief of neurosurgery, chief of gynecological surgery, and a senior urologist.

OR RTP was implemented on April 6, 2015. The reinforcement of RTP awareness included daily OR nursing staff rounds, the implementation of OR nursing staff RTP audits, bi-monthly anesthesia staff meetings, and the availability of table protractors (750 Pitch and Slope Locator; Johnson Level & Tool Mfg. Co., Inc., Mequon, WI) for each OR. The principal recommendation for the OR nursing staff was that RTP should be used when clinically reasonable and technically feasible. The primary recommendations made during discussions with the anesthesia staff were that RTP should be used when clinically reasonable and technically feasible, during tracheal intubation, tracheal extubation, and the operative procedure. The OR nursing staff RTP audits were noncompulsory, performed daily, and they indicated the patient’s position during the operative procedure: (a) persistent RTP, (b) intermittent RTP, or (c) no RTP. The OR nurse completed the audit form at the end of the surgical procedure. A designated general surgical resident, an orthopedic attending surgeon, and a certified registered nurse anesthetist agreed to foster RTP awareness among their peers. Signs were placed outside of each OR as a reminder that RTP may reduce POH.

### RTP surveys

In early 2016 before the study results were released, the OR nursing and anesthesiology staff were given a survey regarding RTP in the OR. The purpose of the survey was to determine the degree to which the staff embraced the notion of RTP. Two statements were included in the OR nursing staff survey: “an intraoperative reverse Trendelenburg position may decrease the risk for postoperative hypoxemia” and “I am inclined to use a reverse Trendelenburg position during the operative procedure when clinically and technically feasible.” Four statements were included in the anesthesiology staff survey: “an intraoperative reverse Trendelenburg position may decrease the risk for postoperative hypoxemia”; “I am inclined to use a reverse Trendelenburg position during tracheal intubation when clinically and technically feasible”; “I am inclined to use a reverse Trendelenburg position during the operative procedure when clinically and technically feasible”; and “I am inclined to use a reverse Trendelenburg position during tracheal extubation when clinically and technically feasible.” Each statement was followed by Likert scale response options: strongly agree, agree, uncertain, disagree, and strongly disagree.

### Cohort analysis organization

Patients from 2012 to 2015 were combined to determine conditions that were associated with mortality, POH, POPA, and the postoperative LOS. Comparisons between 2012 and 2015 represented periods where RTP awareness was negligible or improved, respectively. Relative to the 2012 cohort, the 2015 cohort was also consecutively obtained, with the same inclusion and exclusion criteria. Some inter-group analyses were performed, based on the 2015 OR nursing staff RTP audits that classified patients who had RTP during the operative procedure.

### Statistical analysis

Data were entered into a Microsoft Excel® 2010 spreadsheet (Microsoft Corp., Redmond, WA) and imported into SAS System for Windows, release 9.2 (SAS Institute Inc., Cary, NC), for statistical analyses. For the continuous variable cohort data, mean and standard deviation was used. Pearson correlation analysis was performed to assess associations between two continuous variables. The Wilcoxon rank-sum test was used to compare continuous data between two groups. Analysis of variance was applied to compare continuous data involving more than two groups. The Fischer’s exact test was used to assess the relationship between two dichotomous variables. Multivariate logistic regression analysis was performed to assess the relationship between a dependent variable that was dichotomous and potential independent variables. Multivariate linear regression analysis was performed to evaluate independent variable relationships with a dependent, continuous variable. The level of significance was considered *p* < 0.05.

## Results

### Patients

Among patients aged 18 years or older who had an operative procedure that required endotracheal intubation and a general anesthetic, Glasgow Coma Scale score ≥ 13, and ASA classification I–IV and who did not require tracheal intubation before emergency department arrival, endotracheal intubation > 24 h before surgery, thoracotomy procedure, any cardiac procedure, or more than one surgery requiring tracheal intubation during the same hospitalization, the rates of preoperative pulmonary instability were 6.0% (32/532) for the 2012 patient cohort and 9.1% (100/1100) for the 2015 cohort (*p* = 0.0332). From May 14, 2012, through July 13, 2012, 500 consecutive surgical patients were included in the original published study. The most recent consecutive cohort included 1000 patients who had undergone an operative intervention from May 13, 2015, through September 20, 2015.

### OR personnel surveys

The OR nursing staff survey was conducted in early June 2016, and it included 41 participants. The anesthesiology staff survey was performed in June 2016, and it included 44 anesthesiologists, certified registered nurse anesthetist staff, and student certified registered nurse anesthetist personnel. Responses to the survey statements indicate that the majority strongly agreed or agreed with the notion that RTP may be useful (Table 1).

**Table 1:** Operating room personnel surveys

	Strongly agree or agree	Uncertain	Disagree or strongly disagree
Nursing staff (*n* = 41)			
RTP may decrease POH	34 (82.9%)		7 (17.1%)
Inclined to use RTP	33 (80.5%)		8 (19.5%)
Anesthesiology staff (*n* = 44)			
RTP may decrease POH	41 (93.2%)	1 (2.3%)	2 (4.5%)
Use RTP during tracheal intubation	41 (93.2%)	1 (2.3%)	2 (4.5%)
Use RTP during the operative procedure	39 (88.6%)	1 (2.3%)	4 (9.1%)
Use RTP during tracheal extubation	39 (88.6%)		5 (11.4%)

*RTP* reverse Trendelenburg, *POH* postoperative hypoxemia

### Noncompulsory RTP audits

The OR nursing staff submitted 387 RTP audit forms throughout the period during which the 1000 patients in 2015 underwent an operation. The RTP audit process had been operational for several weeks before the study phase. See LOS associations (within 2015 comparison) for results.

### Mortality associations (combined 2012 and 2015)

From the combined 2012 and 2015 patient cohorts, the mortality rate was 0.6% (9/1500). Hypothesis-generating associations regarding mortality are depicted in Table 2. Compared to non-dying patients, dying patients had increased POH and POPA, older age, higher ASA classification, and a higher rate of emergency operations.

**Table 2:** Mortality associations

	Lived	Died	*p* value
Patients	1491 (99.4%)	9 (0.6%)	
Adverse outcomes			
Postoperative hypoxemia	302 (20.3%)	7 (77.8%)	0.0004
Perioperative aspiration	48 (3.2%)	4 (44.4%)	< 0.0001
Postoperative length of stay (days)	2.5 ± 3.4	5.1 ± 5.7	0.2099
Preoperative risk			
Age	56 ± 17	75 ± 14	0.0016
ASA classification	2.8 ± 0.6	3.2 ± 0.7	0.0464
Emergency procedures	120 (8.1%)	3 (33.3%)	0.0058
Preexisting lung disease	221 (14.8%)	1 (11.1%)	0.7546
Weight (kg)	86 ± 24	80 ± 22	0.4037
Intraoperative risk			
Operating room minutes	128 ± 74	149 ± 69	0.3972
Glycopyrrolate on induction	366 (24.6%)	1 (11.1%)	0.3499
Fluid input (L)	1.6 ± 1.0	2.2 ± 2.0	0.3964

*ASA* American Society of Anesthesiologists

### POH associations (combined 2012 and 2015)

From the combined 2012 and 2015 patient cohorts, the POH rate was 20.6% (309/1500). Hypothesis-generating associations regarding POH are shown in Table 3. Compared to patients without POH, those with POH had increased mortality and LOS, older age, higher ASA classification, higher rate of emergency operations and PELD, higher weight, longer time in the OR, and they received less glycopyrrolate and more fluids. POH rates, according to the surgical procedure category, are depicted in Table 4. Open aortic, cranial, laparotomy, and non-spinal neck procedures had the highest POH rates, and together they define a high-risk POH group. The POH rate was greater for the POH high-risk group (41.3% [107/259]) than for the lower-risk cohort (16.3% [202/1241]; *p* < 0.0001; relative risk 2.5).

**Table 3:** Postoperative hypoxemia (POH) associations

	POH (No)	POH (Yes)	*p* value
Patients	1191 (79.4%)	309 (20.6%)	
Adverse outcomes			
Mortality	2 (0.2%)	7 (2.3%)	0.0004
Postoperative length of stay (days)	2.0 ± 2.6	4.6 ± 4.8	< 0.0001
Preoperative risk			
Age	55 ± 18	63 ± 15	< 0.0001
ASA classification	2.8 ± 0.6	3.1 ± 0.5	< 0.0001
ASA classification 3 or 4	857 (72.0%)	273 (88.4%)	< 0.0001
Emergency procedures	88 (7.4%)	35 (11.3%)	0.0246
Preexisting lung disease	160 (13.4%)	62 (20.1%)	0.0034
Weight (kg)	86 ± 24	89 ± 23	0.0489
Intraoperative risk			
Operating room minutes	122 ± 68	154 ± 90	< 0.0001
Glycopyrrolate on induction	303 (25.4%)	64 (20.7%)	0.0849
Fluid input (L)	1.5 ± 0.9	2.0 ± 1.5	< 0.0001

*ASA* American Society of Anesthesiologists

**Table 4:** POH and POPA rates according to the surgical procedural categories

Procedure	Total	POH	POPA	LOS
Open aortic	17	58.8%	29.4%	3.9
Cranial	55	41.8%	5.5%	4.9
Laparotomy	110	40.9%	11.8%	5.2
Neck	77	37.7%	2.6%	1.6
Spine	285	19.3%	3.5%	2.6
Extremity/pelvis	387	19.1%	2.1%	3.3
Laparoscopy	275	16.0%	2.9%	1.5
Miscellaneous	126	15.9%	1.6%	1.7
Face soft tissue	21	9.5%	0%	1.0
Breast	76	5.5%	0%	0.3
Oral	71	4.2%	1.4%	0.4
	1500	Mean = 20.6%	Mean = 3.5%	Mean = 2.5 days

*POH* postoperative hypoxemia, *POPA* perioperative pulmonary aspiration, *LOS* postoperative length of stay

### POH associations (2012 versus 2015)

A comparison of the 2012 and 2015 patient cohorts is shown in Table 5, and it demonstrates a reduction in 2015 POH. Compared to the 2012 patient cohort, the 2015 patient cohort had a lower POH rate, higher age and ASA classification, and they received less fluid. Results of the multivariate logistic regression analysis of the 1500 patients inferred that POH was likely simultaneously (independently) increased with greater time in the OR (*p* < 0.0001), age (*p* < 0.0001), ASA classification (*p* < 0.0001), and weight (*p* = 0.0078), and decreased for the year 2015 (*p* < 0.0001), and might be decreased with glycopyrrolate administration (*p* = 0.0756). A model equation was created, using the 2012 logistic regression maximum likelihood estimates to predict the POH rate. Using age 54 and ASA classification 2.8 (2012), the POH rate was predicted to be 23.9%, whereas applying age 57 and ASA classification 2.9 (2015), the predicted POH rate was 25.8%. The difference in the POH rate between 2012 and 2015 was 7.5% (25.6% [−] 18.1%). The relative risk reduction in POH with increased RTP sensitivity (2015) was 29.3% (7.5%/25.6%).

**Table 5:** Comparison of postoperative hypoxemia rates for the 2012 and 2015 patient cohorts

	2012	2015	*p* value
Patients	500	1000	
Postoperative hypoxemia	128 (25.6%)	181 (18.1%)	0.0007
Preoperative risk			
Age	54 ± 17	57 ± 17	0.0021
Age ≥ 75 years	62 (12.4%)	186 (18.6%)	0.0023
ASA classification	2.8 ± 0.7	2.9 ± 0.6	0.0323
ASA classification 3 or 4	359 (71.8%)	771 (77.1%)	0.0248
Emergency procedures	36 (7.2%)	87 (8.7%)	0.3182
Preexisting lung disease	69 (13.8%)	153 (15.3%)	0.4406
Weight (kg)	86 ± 24	86 ± 24	0.9357
Intraoperative risk			
Operating room minutes	129 ± 77	128 ± 73	0.8031
Glycopyrrolate on induction	119 (23.8%)	248 (24.8%)	0.6711
Fluid input (L)	1.8 ± 1.2	1.5 ± 1.0	0.0001

*ASA* American Society of Anesthesiologists

### POPA associations (combined 2012 and 2015)

From the combined 2012 and 2015 patient cohorts, the POPA rate was 3.5% (52/1500). Hypothesis-generating associations regarding POPA are depicted in Table 6. Compared to patients without POPA, those with POPA had an increased mortality and LOS, older age, higher ASA classification, rate of emergencies, and PELD, and longer time in the OR and they received more fluids.

**Table 6:** Perioperative pulmonary aspiration (POPA) associations

	POPA (No)	POPA (Yes)	*p* value
Patients	1448 (96.5%)	52 (3.5%) 95% CI: 2.7–4.5%	
Adverse outcomes			
Mortality	5 (0.4%)	4 (7.7%)	0.0004
Postoperative length of stay (days)	2.3 ± 3.0	8.8 ± 6.7	< 0.0001
Preoperative risk			
Age	60 ± 17	64 ± 18	0.0012
Age ≥ 75 years	230 (15.9%)	18 (34.6%)	0.0004
ASA classification	2.8 ± 0.6	3.1 ± 0.6	0.0004
ASA classification 3 or 4	1084 (74.9%)	46 (88.5%)	0.0254
Emergency procedures	113 (7.8%)	10 (19.2%)	0.0032
Preexisting lung disease	210 (14.5%)	12 (23.1%)	0.0871
Weight (kg)	86 ± 24	82 ± 24	0.1397
Intraoperative risk			
Operating room minutes	126 ± 71	190 ± 114	0.0002
Glycopyrrolate on induction	354 (24.5%)	13 (25.0%)	0.9274
Fluid input (L)	1.6 ± 0.9	2.9 ± 2.4	0.0003

*ASA* American Society of Anesthesiologists, *CI* confidence interval

Open aortic, cranial, and laparotomy procedures had the highest POPA rates and together they define a high-risk POPA group (Table 4). The POPA rate was greater for the POPA high-risk group (11.5% [21/182]) than for the lower-risk cohort (2.4% [31/1318]; *p* < 0.0001; relative risk 4.8).

### POPA associations (2012 versus 2015)

The POPA rate was lower for the 2015 patient cohort (2.8% [28/1000]) than for the 2012 patient cohort (4.8% [24/500]; *p* = 0.0459). Of 28 patients with POPA in 2015, 26 had POH and the other 2 had intraoperative hypoxemia. Of 24 patients with POPA in 2012, 20 had POH and the other 4 had intraoperative hypoxemia. Of 283 patients with IOH or POH in 2015, 81 (28.6%) underwent thoracic imaging, whereas of 150 patients with IOH or POH in 2012, 50 (33.3%) underwent thoracic imaging (*p* = 0.3098). Results of multivariate logistic regression analysis suggested that POPA was simultaneously (independently) increased with greater time in the OR (*p* < 0.0001), higher age (*p* = 0.0088), and emergency procedures (*p* = 0.0001), and decreased with the year 2015 (*p* = 0.0171), and possibly increased with higher ASA classification (*p* = 0.0623).

A comparison of the 2015 patient cohort (documented to be in RTP throughout all or a portion of the operative procedure) and 2012 cohort (decreased RTP) is depicted in Table 7. The two groups are reasonably well matched for preoperative and intraoperative risk criteria. However, the operative procedure RTP group had a substantial reduction of POPA. Findings of multivariate logistic regression analysis implied that POPA was likely simultaneously (independently) increased with emergency procedures (*p* = 0.0452) and PELD (*p* = 0.0289) and decreased with documented operative procedure RTP in 2015 (*p* = 0.0290).

**Table 7:** Perioperative aspiration comparison of 2015 cohort with documented procedure RTP to 2012 cohort

	RTP (↓) (2012)	RTP (Yes) (2015)	*p* value
Total	500 (74.1%)	175 (25.9%)	
Perioperative aspiration	24 (4.8%)	1 (0.6%)	0.0088
Preoperative risk			
Age	54 ± 17	56 ± 17	0.2310
ASA classification	2.8 ± 0.6	2.9 ± 0.6	0.822
Emergency procedures	36 (7.2%)	5 (2.9%)	0.0384
Preexisting lung disease	69 (13.8%)	41 (23.4%)	0.0030
Weight (kg)	86 ± 24	89 ± 23	0.1571
Intraoperative risk			
Operating room minutes	129 ± 77	126 ± 61	0.5225
Glycopyrrolate on induction	119 (23.8%)	48 (27.4%)	0.3384
Fluid input (L)	1.8 ± 1.2	1.6 ± 0.9	0.0184

*RTP* reverse Trendelenburg, **↓** decreased RTP, *Yes* RTP throughout or a portion of the operative procedure, *ASA* American Society of Anesthesiologists

### LOS associations (combined 2012 and 2015)

From the combined 2012 and 2015 patient cohorts, the mean LOS was 2.5 ± 3.4 days (range 0–37 days). Of 1500 patients, 76.6% (*n* = 1149) were discharged by the third postoperative day. Hypothesis-generating associations regarding LOS are depicted in Table 8. The data inferred that an increased LOS was likely related to POH, POPA, time in the OR, age, ASA classification, emergency status, fluid input, and POH high-risk procedures (i.e., open aortic, cranial, laparotomy, and non-spinal neck procedures). The data suggested that a decreased LOS was associated with glycopyrrolate administration upon induction.

**Table 8:** Postoperative length of stay (LOS) associations

	LOS (days)	*R* value	*p* value
POH, no	2.0 ± 2.6		
POH, yes	4.6 ± 7.8		< 0.0001
Perioperative aspiration, no	2.3 ± 3.0		
Perioperative aspiration, yes	8.8 ± 6.7		< 0.0001
Time in operating room		(+) 0.25	< 0.0001
< 150 min	2.1 ± 3.1		
≥ 150 min	3.5 ± 3.7		< 0.0001
Age		(+) 0.20	< 0.0001
< 75 years	2.3 ± 3.1		
≥ 75 years	3.6 ± 4.5		< 0.0001
ASA classification		(+) 0.20	< 0.0001
1 or 2	1.6 ± 2.1		
3 or 4	2.8 ± 3.6		< 0.0001
Elective procedures	2.3 ± 3.2		
Emergency procedures	4.6 ± 4.6		< 0.0001
Glycopyrrolate on induction, no	2.7 ± 3.6		
Glycopyrrolate on induction, yes	1.9 ± 2.3		< 0.0001
Fluid input (L)		(+) 0.25	< 0.0001
< 2.0 L	2.1 ± 3.1		
≥ 2.0 L	3.6 ± 3.8		< 0.0001
POH high risk, no	2.2 ± 3.0		
POH high risk, yes	4.0 ± 4.4		< 0.0001

*ASA* American Society of Anesthesiologists, *POH* postoperative hypoxemia, *high risk*, open aortic, cranial, laparotomy, and non-spinal neck operative procedures

### LOS associations (2012 versus 2015)

Despite the decreased POH rate for 2015, the LOS for 2015 (2.6 ± 3.4 days) was similar to that in 2012 (2.3 ± 3.3 days; *p* = 0.0714). Results of multivariate linear regression analysis (combined 2012 and 2015 cohorts) implied that LOS was likely to be simultaneously (independently) increased with increasing age (*p* < 0.0001) and ASA classification (*p* < 0.0001), but it was not different according to the year (*p* = 0. 2737).

### LOS associations (within 2015 comparison)

The 2015 audit patient categorizations were no RTP, 212 (21.2%) patients; RTP throughout the operative procedure, 64 (6.4%); RTP during a portion of the operative procedure, 111 (11.1%); and no audit, 613 (61.3%). The durations of LOS (days) by patient categorization were no RTP, 2.4 ± 2.8; RTP throughout the operative procedure, 1.8 ± 2.6; RTP during a portion of the operative procedure, 2.4 ± 3.0; and no audit, 2.8 ± 3.7 (*p* = 0.0533).

The four subsets were reorganized into two groups: RTP–Yes (RTP throughout or a portion of the operative procedure) and RTP–No (no RTP or no audit) (Table 9). The RTP–Yes group had fewer emergency procedures but a greater weight and more instances of PELD than the RTP–No group. Findings of univariate analysis suggested that compared to the RTP–No group, the RTP–Yes group had a reduction in postoperative LOS. Results of multivariate linear regression analysis inferred that LOS was likely to simultaneously (independently) increase with the ASA classification (*p* < 0.0001) and decrease with glycopyrrolate administration (*p* = 0.0002) and operative procedure RTP (*p* = 0.0399).

**Table 9:** Outcomes according to operative procedure reverse Trendelenburg (RTP) status

Audit	RTP (No)	RTP (Yes)	*p* value
	825 (82.5%)	175 (17.5%)	
Postoperative length of stay (days)	2.7 ± 3.5	2.2 ± 2.8	0.0246
Preoperative risk			
Age	57 ± 18	56 ± 17	0.3479
ASA classification	2.8 ± 0.6	2.9 ± 0.6	0.5627
Emergency procedures	82 (9.9%)	5 (3.0%)	0.0025
Preexisting lung disease	112 (13.6%)	41 (23.4%)	0.0010
Weight (kg)	86 ± 24	89 ± 23	0.0746
Intraoperative risk			
Operating room minutes	129 ± 75	126 ± 61	0.5419
Glycopyrrolate on induction	200 (24.2%)	48 (27.4%)	0.3753
Fluid input (L)	1.5 ± 1.0	1.6 ± 0.9	0.5296

*No* no operative RTP or no audit, *Yes* RTP throughout or a portion of the operative procedure, *ASA* American Society of Anesthesiologists

## Discussion

### Evidence for increased RTP during 2015

Before and during the study period, multiple processes were implemented to foster the use of RTP. The director of surgical and perioperative services, manager and assistant manager of the OR nursing staff, and chief of anesthesiology endorsed the notion that RTP may be beneficial for patients undergoing general anesthesia. Multiple in-service activities were conducted for the OR nursing and anesthesiology staff to encourage the use of RTP, when clinically and technically feasible. Additionally, we gave presentations to and had discussions with surgeons of multiple disciplines regarding the potential benefit of RTP. Daily OR nursing staff rounds were performed to reinforce the use of RTP during the operative procedure. OR table protractors were made available for each room to facilitate RTP and prompt the use of RTP. Signs were placed outside of each OR, as a reminder that RTP may reduce POH. Daily audits served to remind the OR nursing staff to use RTP.

According to the survey results taken before the study results were released, substantial evidence exists that the OR nursing and anesthesiology staff embraced the notion that RTP may be important. Numerous discussions between the OR nurse manager and assistant manager with OR nursing staff typically indicated an understanding by the staff that RTP may be of value. These conversations were supported by the OR nursing staff survey. Several dialogs between the chief of anesthesiology and the anesthesia staff usually indicated an understanding that RTP may be of value. These responses are shown in the anesthesiology staff survey results.

The OR nursing staff audits specifically document the use of RTP during 2015. Similarly, the OR nurse manager and assistant manager observed the use of RTP on numerous occasions during their OR rounds and throughout the day. The managers confirmed that RTP was more commonly used in 2015 than in 2012. The chief of anesthesiology found that RTP during the OR procedure was common, and it was typical for tracheal intubation and extubation. She has an unequivocal sense that RTP was more frequently used in 2015 than in 2012. Importantly, the POH rate in 2015 was substantially decreased, despite the higher ASA classification and age. The increased use of RTP offers a plausible explanation for decreased POH and POPA rates in 2015. On the basis of the aforementioned findings, we conclude that RTP is associated with improved perioperative outcomes. The 2015 audit, which showed that patients with documented RTP had a decrease in LOS, supports this argument.

### OR personnel surveys

After the study period, the anesthesia and OR nursing staff were surveyed regarding their perceptions of RTP. The surveys were conducted before data dissemination to mitigate any bias that may have been fostered by having knowledge of the study results. The intent of the survey was to reveal staff attitudes toward RTP during the study period. Of 85 anesthesia and OR nursing staff members participating in the surveys, more than 75% indicated that they perceived RTP as beneficial and that it should be used when feasible. Disagreement or uncertainty responses were sparse; however, dissenting replies suggest that individual perceptions were volitional and not biased by peer pressure. Results of the surveys indicate that the staff members were receptive to implementing RTP, under appropriate circumstances, during the study period.

### Noncompulsory RTP audits

During 2015, nearly 40% of the study patients had a noncompulsory RTP audit completed throughout the study period. The audit indicated whether RTP was used during the surgical procedure (all the time, some of the time, or none of the time). However, it did not indicate whether RTP was used during tracheal intubation or extubation. Importantly, the RTP audit process served as a daily reinforcement that RTP may be beneficial and that it should be considered when appropriate.

### Mortality associations (combined 2012 and 2015)

Compared to non-dying patients, dying patients had higher rates of POH, POPA, and emergency surgery and greater age and ASA classification. The higher rates of POH and POPA in dying patients suggest the notion that these outcomes, in particular, and pulmonary complications, in general, are likely to be clinically valid conditions and have an association with adverse outcomes. These findings also propose that the abstraction processes used in this study to categorize patients with POH and POPA may be clinically useful. The higher rate of emergency surgery procedures, older age, and higher ASA classification in non-survivors suggest that these preoperative risk conditions have a relationship with mortality.

### POH associations (combined 2012 and 2015)

When the 2012 and 2015 patient cohorts were combined, POH was shown to likely be associated with adverse postoperative outcomes, and preoperative and intraoperative risk conditions. The potential association of POH with increased postoperative mortality and longer LOS indicates the need to substantiate the clinical validity of POH as an entity, and it suggests the credibility of our particular medical record abstraction process. The relationship between POH and intraoperative risk conditions suggests that OR events are linked to the development of POH. Additionally, the data imply that glycopyrrolate administration on anesthesia induction and shortening the surgical duration might be associated with improved outcomes. Further, the hypothesis-generating association of POH with preoperative risk factors suggests that host conditions may affect subsequent POH; however, these are typically not modifiable. The POH rates were substantially increased with open aortic, cranial, laparotomy, and non-spinal neck surgical procedures, compared to other procedural categories. This finding infers that these surgical procedures may need to receive more attention, especially when considering future strategies to mitigate POH. Patients with preoperative SpO_2_ < 94% were excluded in order to enhance the likelihood that hypoxemia, if it were to develop, would have been related to intraoperative risk conditions.

### POH associations (2012 versus 2015)

Compared to 2012, the POH rate in 2015 appeared to be substantially decreased, despite a higher age and ASA classification for the 2015 patient cohort. The time in the OR, patient weight, emergency procedures, glycopyrrolate administration, and PELD rates were comparable for the 2012 and 2015 patient cohorts. Findings of multivariate logistic regression analysis suggest that POH is simultaneously (independently) reduced for the year 2015, i.e., the period with increased use of RTP, and might be reduced with glycopyrrolate administration on anesthesia induction. Using the POH prediction model developed from the 2012 patient cohort, the POH rate in 2015 was predicted to be higher due to the higher ASA classification and age. However, the POH rate was significantly decreased during 2015, inferring that there is likely to be an inverse association between POH and RTP. The POH relative risk reduction was nearly 30% for the 2015 patient cohort, compared to the 2012 cohort.

### POPA associations (combined 2012 and 2015)

When the 2012 and 2015 cohorts were combined, POPA was shown to likely be associated with adverse postoperative outcomes, and preoperative and intraoperative risk conditions. The hypothesis-generating association of POPA with increased postoperative mortality and longer LOS indicates the need to substantiate the clinical validity of POPA as an entity and to confirm the credibility of our specific medical record abstraction process in a prospective study. The relationship between POPA and intraoperative risk conditions suggests that OR events are linked to the development of POPA. Furthermore, the potential association of POPA with preoperative risk factors suggests that host conditions may affect subsequent POPA; however, these are usually not modifiable. The POPA rates were substantially increased with open aortic, cranial, and laparotomy surgical procedures compared to other procedural categories. These surgical procedures may need to receive greater consideration, when contemplating potential strategies to mitigate POPA.

### POPA associations (2012 versus 2015)

The POPA rate in 2015 compared to that in 2012 was significantly decreased. This decrease occurred despite a higher age and ASA for the 2015 patient cohort, factors that are likely to be associated with an increased risk for POPA. Results of multivariate logistic regression analysis suggested that POPA was simultaneously (independently) reduced for the year 2015, i.e., the period with increased use of RTP. Using the OR nursing staff audits for 2015, patients with documented RTP anytime during the surgical procedure had a reduction in POPA. This finding was substantiated by results of univariate and multivariate statistical analyses. In summary, these findings imply that RTP is likely to be associated with a reduction in POPA.

### LOS associations (combined 2012 and 2015)

The mean LOS was 2.5 days, with 75% of patients having been discharged by the third postoperative day. After combining the 2012 and 2015 cohorts, LOS was shown to likely be associated with adverse postoperative outcomes, and preoperative and intraoperative risk conditions. As previously stated, POH and POPA appear to have been related to an increased LOS. Patients undergoing high-risk POH procedures (i.e., open aortic, cranial, laparotomy, non-spinal neck procedures) compared to other procedures had a substantially longer LOS. LOS was longer with certain host conditions (increased age and ASA classification and emergency procedures). The following intraoperative conditions are likely to be associated with increased LOS: increased OR time, no glycopyrrolate administration, and more intraoperative fluid administration. These data suggest that administering glycopyrrolate on anesthesia induction and decreasing the duration of the surgical procedure might be useful strategies for decreasing LOS.

### LOS associations (2012 versus 2015)

Despite the decreased POH rate for 2015, the LOS was similar for 2015 and 2012. A potential reduction in LOS due to the decrease in the lower POH rate for 2015 was likely offset by the higher age and ASA classification for 2015, as these factors appear to be associated with an increase in LOS.

### LOS associations (within 2015 comparison)

Using the OR nursing staff audits for 2015, patients with documented RTP during the surgical procedure had a reduced LOS. This finding is supported by the univariate and multivariate statistical analyses. Specifically, the group with documented RTP likely had fewer emergency procedures but a greater weight and higher PELD rate. Results of multivariate linear regression analysis suggested that LOS was simultaneously (independently) decreased with glycopyrrolate administration on anesthesia induction and with documented RTP during the operative procedure. These data infer that glycopyrrolate administration and RTP are likely strategies to reduce LOS in patients undergoing general anesthesia and endotracheal intubation.

### Study limitations

The glycopyrrolate associations with POH had *p* values of 0.05–0.09 suggesting that subsequent studies may or may not demonstrate an inverse association with POH. Certainly, our investigation may not have been sufficiently powered. The primary study limitation is the failure to document RTP use for every surgical case during 2012 and 2015. Ideally, there would be documentation for all 1500 patients regarding the use or non-use of RTP during tracheal intubation, the surgical procedure, and tracheal extubation. This would have enabled us to precisely and objectively determine RTP use in the OR during 2012 and 2015. Additionally, RTP as a mechanism to reduce POH and POPA was not studied in a randomized, controlled manner. Other factors that may have affected outcomes but were not investigated include the intraoperative tidal volume, positive end-expiratory pressure, tracheal cuff pressure levels, intraoperative body position, volume of secretions aspirated before extubation, and amounts of postoperative narcotic patients received.

## Conclusions

This study’s findings are hypothesis-generating, not hypothesis-confirming. A randomized clinical trial is needed to confirm (1) whether POH and POPA have an association with mortality and postoperative LOS, (2) if RTP has an inverse association with POH, POPA, and LOS, (3) whether glycopyrrolate administration on anesthesia induction is associated with a lower postoperative LOS and a reduction in POH, and (4) if our particular medical record abstraction process is credible. POH rates, POPA rates, and postoperative LOS are likely to be substantially increased with open aortic, cranial, laparotomy, and non-spinal neck surgical procedures compared to other procedural categories. The study results signal the need to confirm whether RTP, glycopyrrolate administration, and a shorter surgery time are beneficial for improving perioperative outcomes in patients undergoing general anesthesia and endotracheal intubation. Heightened attention may need to be paid to surgical procedures with an increased risk for POH, POPA, and prolonged LOS, when considering future strategies to mitigate pulmonary adverse outcomes. We recommend that future investigations have a prospective design and ideally include group randomization.

## Abbreviations

ASA: American Society of Anesthesiology
EMR: Electronic medical record
IOH: Intraoperative hypoxemia
LOS: Postoperative length of stay
OR: Operating room
PELD: Preexisting lung disease
POH: Postoperative hypoxemia
POPA: Perioperative pulmonary aspiration
RTP: Reverse Trendelenburg positioning
SpO_2_: Oxygen saturation as measured by pulse oximetry
